# Supplementary material for: Key phosphorylation sites for robust β-arrestin2 binding at the MOR revisited
Source: Commun Biol. 2024 Aug 2;7:933. doi: 10.1038/s42003-024-06571-1 (PMC11297201; doi:10.1038/s42003-024-06571-1)
Supplement: Supplementary file 2 — Supplementary Information [file 42003_2024_6571_MOESM2_ESM.pdf]

Supplementary methods

mGsi recruitment BRET

Control HEK293 cells were seeded in 10-cm cell culture dishes and transfected with 4 µg mGsi-Venus and 1 µg Flag-MOR-NLuc (or STANT-2A mutant) using a 1:6 DNA:PEI ratio. 24h after transfection cells were harvested and transferred into white 96-well Poly-D-Lysine coated CulturPlates (PerkinElmer) in DMEM + 10% FBS. 48h post-transfection, the media in each well was aspirated, washed with Hank's balanced salt solution pH 7.4 (HBSS), replaced with HBSS and then kept at 37 °C for the remainder of the assay. Cells were then treated with 10 µL of ligand at 10x final concentration and incubated for 5 minutes, followed by addition of 10 µL furimazine (final concentration 5 µM) and incubated for 5 minutes. Plates were then read on a PHERAstar Plate Reader (BMG LABTECH, Ortenberg, Germany) using the BRET1 filter set (535 ± 30 nm (fluorescence), 475 ± 30 nm luminescence). Raw BRET signals were calculated as the emission intensity at 520–545 nm divided by the emission intensity at 475–495 nm, and the vehicle-subtracted BRET ratio (drug-induced increase in BRET) was calculated and plotted.

Quantification of HA-MOR expression in knock-out cell lines

To determine MOR expression levels in the different cell lines, we performed the 7TM phosphorylation assay as previously described (Kaufmann et al., 2022). Control, ΔGRK2/3, ΔGRK5/6 and ΔQ-GRK cells were seeded into a poly-L-lysine-coated 96-F-bottom-well cell culture microplate (Greiner Bio-One, 655180). When a confluence of >95% was reached, cells were lysed in 100 µl per well detergent buffer (150 mM NaCl; 50 mM Tris-HCl, pH 7.4; 5 mM EDTA; 1% Igepal CA-360; 0.5% deoxycholic acid; 0.1% SDS). After centrifugation, 20 µl cell lysate were transferred to a 96-U-bottom-well assay plate (Greiner Bio-One, 650101) and HA-tagged receptors were enriched using mouse anti-HA magnetic beads (Thermo Fisher, 88837). Beads were washed with PBS containing 0.1% Tween20®. The np-MOP antibody (7TM Antibodies, 7TM0319N) which binds to the C-terminal region of the receptor independently of its phosphorylation status was used as primary antibody and an HRP-linked anti-rabbit antibody (Cell Signaling Technology) was used as secondary antibody. With the addition of a substrate solution (Super AquaBlue detection solution, Thermo Fisher, 00-4203-58), a colorimetric reaction was induced and subsequently stopped with 0.625 M oxalic acid. Through a handheld magnetic block (V&P Scientific 771HH-H/Millipore, 40-285), beads were separated, and the supernatant was transferred into a 96-well F-bottom

detection plate (Greiner Bio-One, 655182). Optical density (OD) at 405 nm was measured at the FlexStation3 microplate reader (Molecular Devices). Data were acquired using the SoftMax Pro 5.4 software and calculations were performed in Excel 16.0. Background signals were subtracted from raw OD values and normalized to Control. For Control cells, the receptor amount per mg membrane protein has been determined using saturation binding assays (Kaufmann et al., 2022). Using this information, we were able to estimate the receptor amount for the ΔGRK cell lines.

References

Kaufmann, J., Blum, N. K., Nagel, F., Schuler, A., Drube, J., Degenhart, C., Engel, J., Eickhoff, J. E., Dasgupta, P., Fritzwanker, S., Guastadisegni, M., Schulte, C., Miess-Tanneberg, E., Maric, H. M., Spetea, M., Kliewer, A., Baumann, M., Klebl, B., Reinscheid, R. K., . . . Schulz, S. (2022). A bead-based GPCR phosphorylation immunoassay for high-throughput ligand profiling and GRK inhibitor screening. *Commun Biol*, *5*(1), 1206. https://doi.org/10.1038/s42003-022-04135-9

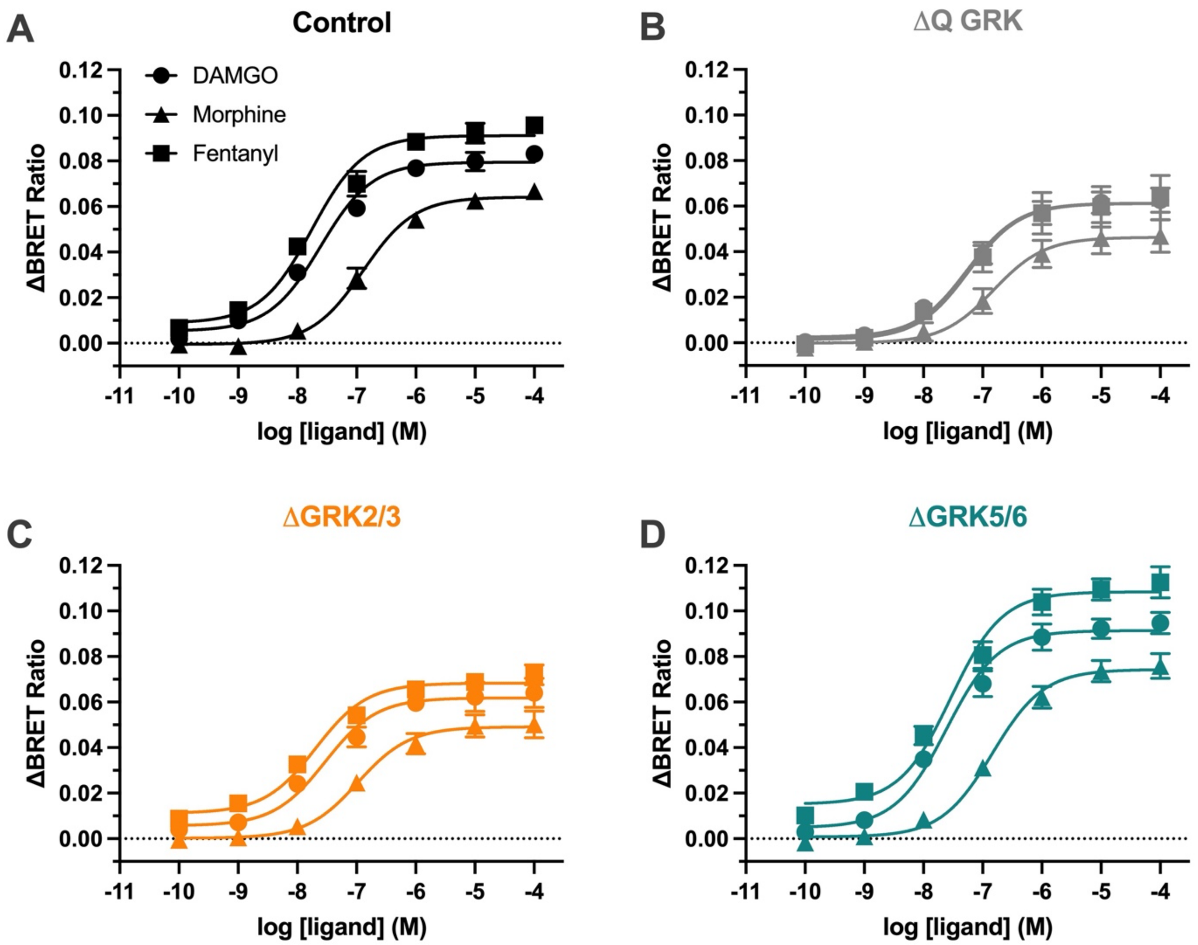

Supplementary Figure 1. mGsi recruitment to the MOR in control and GRK knockout cells. mGsi-Venus recruitment to the Flag-mMOR-Nluc upon stimulation with DAMGO, fentanyl or morphine for 10 min at 37ºC in **(A)** control cells, **(B)** quadruple (ΔQ-GRK) or subfamily **(C)** ΔGRK2/3 or **(D)** ΔGRK5/6 knockout cells. Data show the mean ± SEM baseline-corrected BRET ratio of 5 independent experiments performed in triplicate.

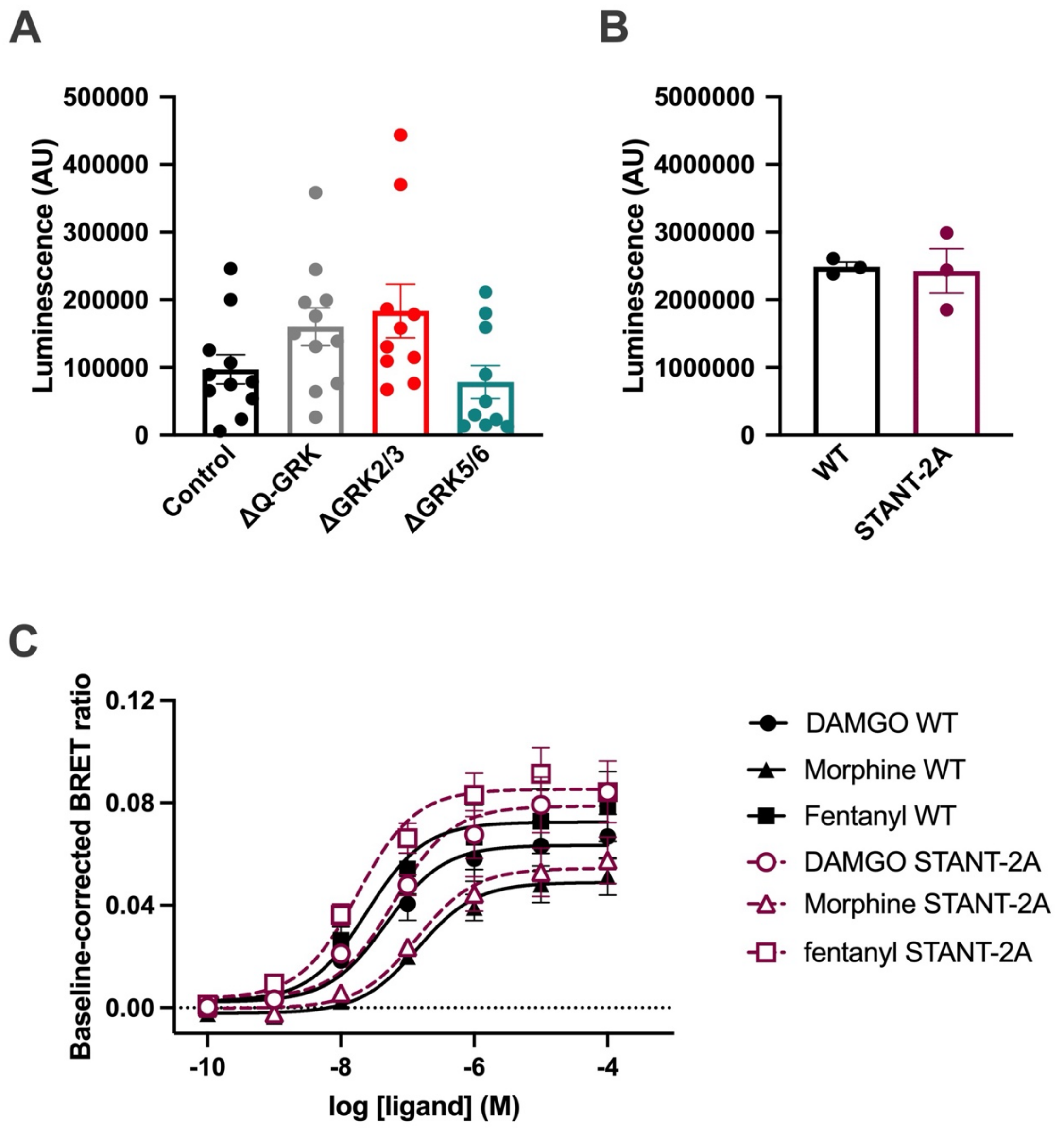

Supplementary Figure 2. MOR WT and STANT-2A expression and STANT-2A function (A) Flag-mMOR-Nluc luminescence in control and GRK knockout cells. Symbols represent individual repeats, bar shows mean ± SEM of 4 independent experiments performed in triplicate **(B)** Luminescence of WT or STANT-2A MOR in control cells. Symbols represent individual repeats, bar shows mean ± SEM of 3 independent experiments performed in triplicate. **(C)** mGsi recruitment BRET to WT and STANT-2A MOR**.** Data show the mean ± SEM BRET ratio of 3 independent experiments performed in triplicate

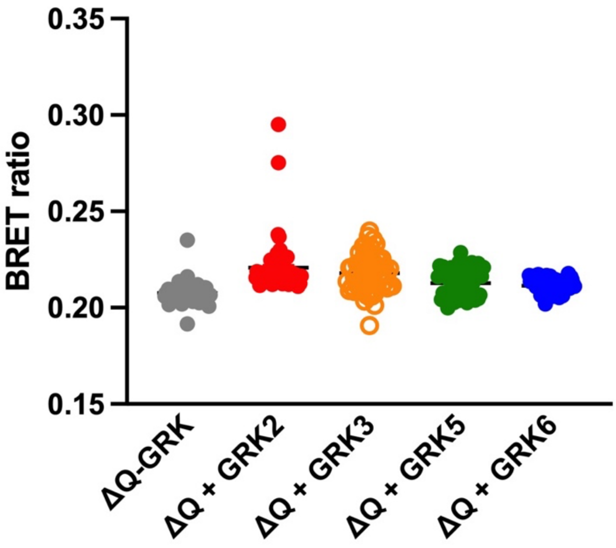

Supplementary Figure 3. Baseline BRET ratio for β-arrestin2 recruitment to the MOR in GRK knockout cells upon individual GRK overexpression. β-arrestin2-Venus / Flag-mMOR-Nluc BRET ratio prior to ligand stimulation was measured in ΔQ-GRK overexpressing GRK2/3/5/6. Data show the individual baseline measurements for each well shown in Figure 3.

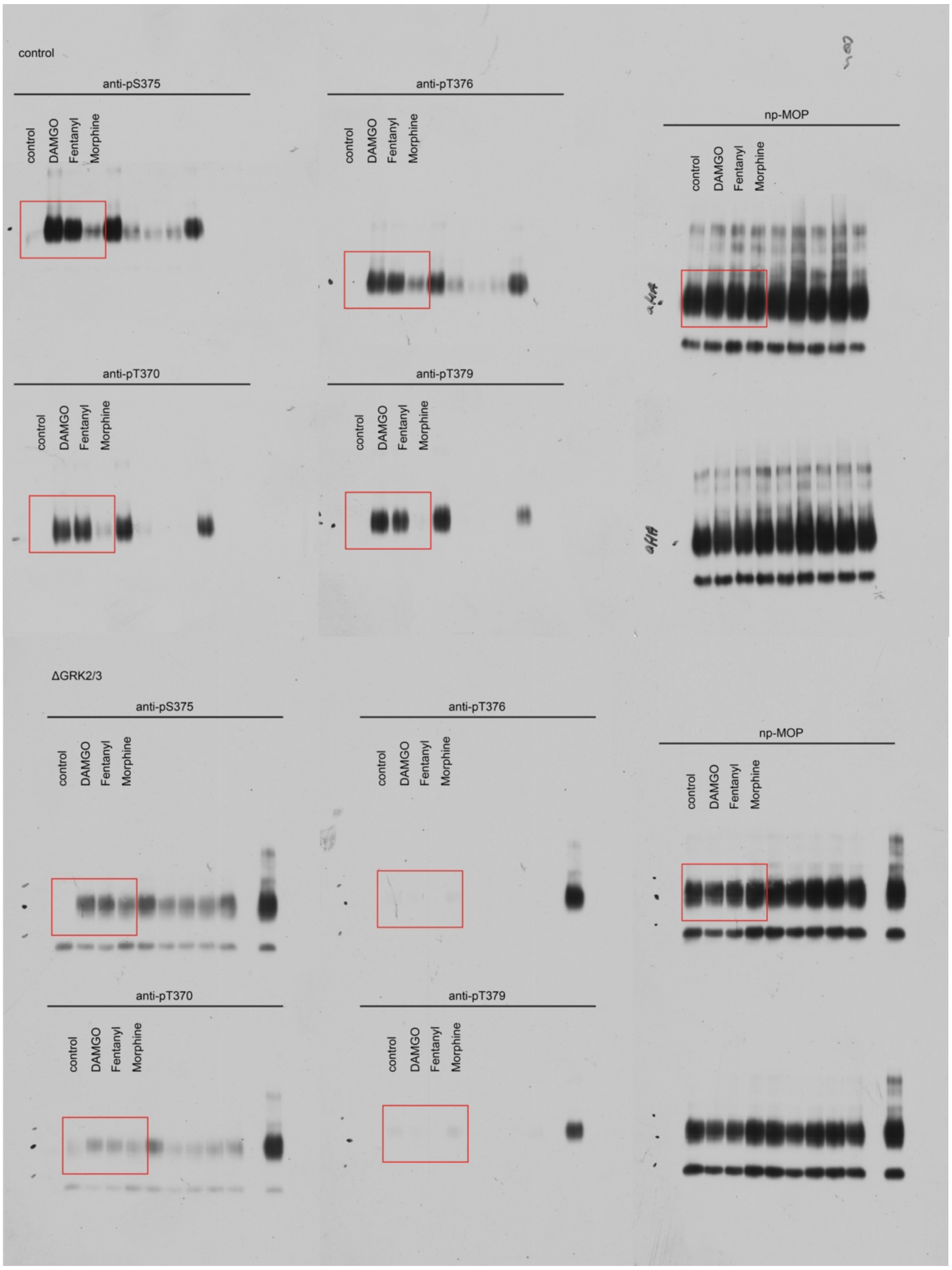
control
anti-pS375
control
DAMGO
Fentanyl
Morphine
anti-pT376
control
DAMGO
Fentanyl
Morphine
np-MOP
control
DAMGO
Fentanyl
Morphine
anti-pT370
control
DAMGO
Fentanyl
Morphine
anti-pT379
control
DAMGO
Fentanyl
Morphine
ΔGRK2/3
anti-pS375
control
DAMGO
Fentanyl
Morphine
anti-pT376
control
DAMGO
Fentanyl
Morphine
np-MOP
control
DAMGO
Fentanyl
Morphine
anti-pT370
control
DAMGO
Fentanyl
Morphine
anti-pT379
control
DAMGO
Fentanyl
Morphine

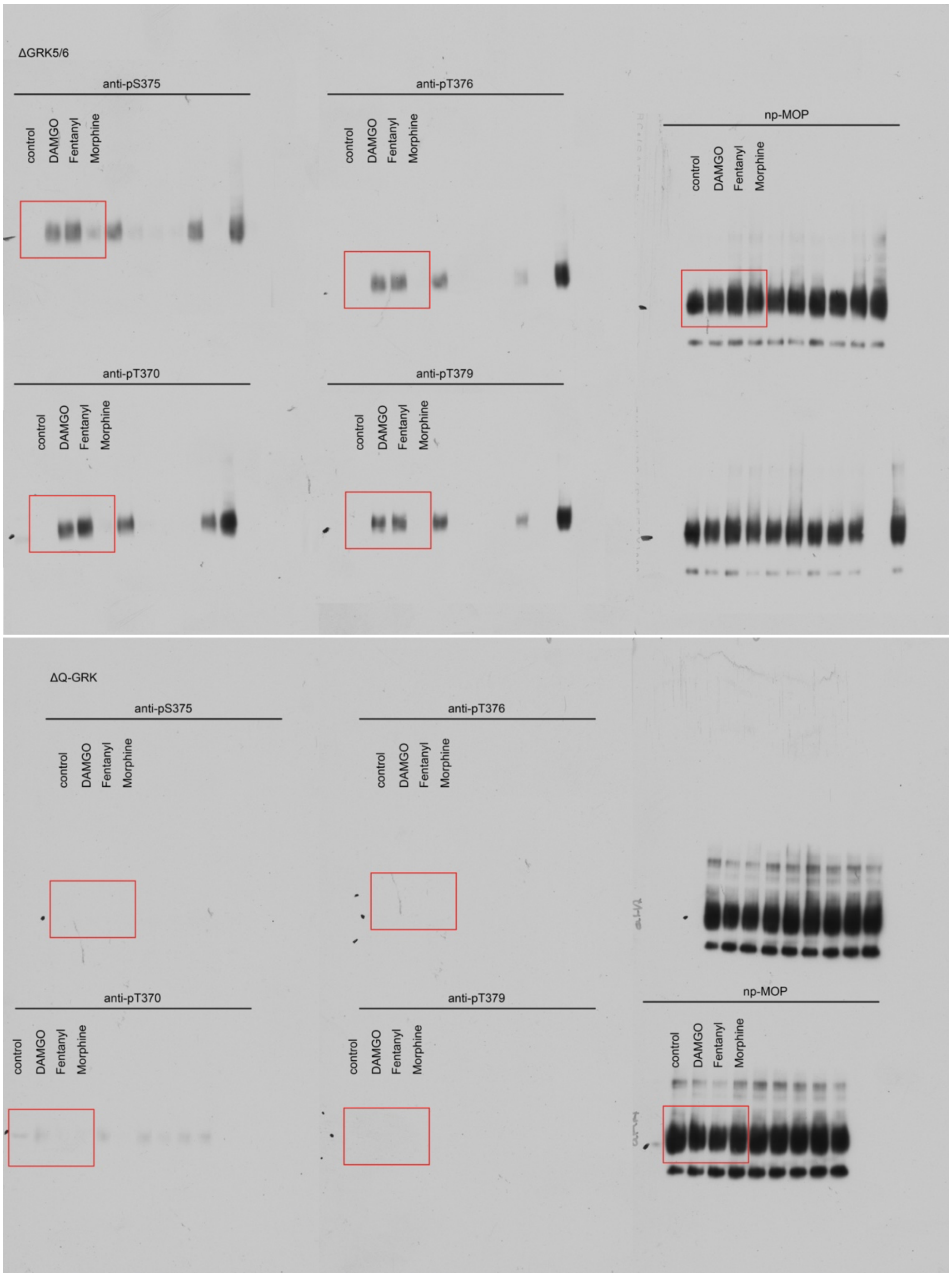

Supplementary Figure 4. Original western blot images of data presented in Figure 1. The red frame marking the section displayed in the figure.

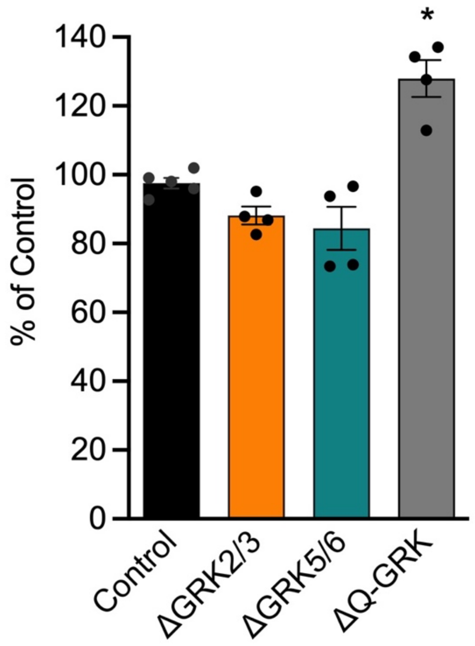

Supplementary Figure 5. Quantification of HA-mMOR expression levels in knock-out cells. Control, ΔGRK2/3, ΔGRK5/6 and ΔQ-GRK cells were treated according to the 7TM phosphorylation assay protocol (see Methods). HA-tagged proteins were enriched, and MOR was detected using a phosphorylation-independent antibody. Data were normalized to optical density of Control cells band. Data represent mean ± SEM of n=4 independent replicates performed in duplicate. * $p<0.05$ compared to control cells, one-way ANOVA ($F(3,12)=20.70$).

Supplementary Table 1. Statistical Analyses for Figure 1C.

Agonist-induced phosphorylation in control cells compared to agonist-induced phosphorylation in ΔGRK cells using one-way-ANOVA				
	**condition**	**p value**		**F**
**pT370-MOP**	Control + DAMGO			
	ΔGRK 2/3 + DAMGO	0.018	*	F (3,8)=8.82, p<0.05
	ΔGRK5/6 + DAMGO	0,161	ns	
	ΔGRKQ + DAMGO	0.003	**	
	Control + Fentanyl			
	ΔGRK2/3 + Fentanyl	0,0003	***	F (3,8)=29.44, p<0.05
	ΔGRK5/6 + Fentanyl	0,0262	*	
	ΔGRKQ + Fentanyl	<0,0001	****	
	Control + Morphine			
	ΔGRK2/3 + Morphine	0,0688	ns	F(3,8)=4.11, p<0.05
	ΔGRK5/6 + Morphine	0,0965	ns	
	ΔGRKQ + Morphine	0,0289	*	
**pS375-MOP**	Control + DAMGO			
	ΔGRK 2/3 + DAMGO	0,1108	ns	F(3,8)=6.21, p<0.05
	ΔGRK5/6 + DAMGO	0,0746	ns	
	ΔGRKQ + DAMGO	0,0069	**	
	Control + Fentanyl			
	ΔGRK2/3 + Fentanyl	0,0211	*	F(3,8)=17.16, p<0.05
	ΔGRK5/6 + Fentanyl	0,0061	**	
	ΔGRKQ + Fentanyl	0,0002	***	
	Control + Morphine			
	ΔGRK2/3 + Morphine	0,1812	ns	F (3,8)=6.86, p<0.05
	ΔGRK5/6 + Morphine	0,0498	*	
	ΔGRKQ + Morphine	0,0145	**	
**pT376-MOP**	Control + DAMGO			
	ΔGRK 2/3 + DAMGO	0,0003	***	F (3,8)=22.63, p<0.05
	ΔGRK5/6 + DAMGO	0,0126	*	
	ΔGRKQ + DAMGO	0,0003	***	
	Control + Fentanyl			
	ΔGRK2/3 + Fentanyl	<0,0001	****	F (3,8)=93.39, p<0.05
	ΔGRK5/6 + Fentanyl	0,0005	***	
	ΔGRKQ + Fentanyl	<0,0001	****	
	Control + Morphine			
	ΔGRK2/3 + Morphine	0,0119	*	F (3,8)=7.35, p<0.05
	ΔGRK5/6 + Morphine	0,0149	*	
	ΔGRKQ + Morphine	0,0115	*	
**pT379-MOP**	Control + DAMGO			
	ΔGRK 2/3 + DAMGO	0,0005	***	F (3,8)=20.32, p<0.05
	ΔGRK5/6 + DAMGO	0,0163	*	
	ΔGRKQ + DAMGO	0,0004	***	
	Control + Fentanyl			
	ΔGRK2/3 + Fentanyl	0,0019	**	F (3,8)=13.07, p<0.05
	ΔGRK5/6 + Fentanyl	0,0488	*	
	ΔGRKQ + Fentanyl	0,0017	**	
	Control + Morphine			
	ΔGRK2/3 + Morphine	0,3183	ns	F (3,8)=1.29, p>0.05
	ΔGRK5/6 + Morphine	0,3142	ns	
	ΔGRKQ + Morphine	0,3149	ns	

Phosphorylation in untreated control cells compared to agonist-induced phosphorylation in control cells using one-way-ANOVA				
	**condition**	**p value**		**F**
**pT370-MOP**	Control untreated			
	Control + DAMGO	0,0122	*	F (3,7)=8.59, p<0.05
	Control + Fentanyl	0,0114	*	
	Control + Morphine	0,4563	ns	
**pS375-MOP**	Control untreated			
	Control + DAMGO	0,0134	*	F (3,7)=6.39, p<0.05
	Control + Fentanyl	0,0146	*	
	Control + Morphine	0,0488	*	
**pT376-MOP**	Control untreated			
	Control + DAMGO	0,0044	**	F (3,7)=11.18, p<0.05
	Control + Fentanyl	0,0082	**	
	Control + Morphine	0,0367	*	
**pT379-MOP**	Control untreated			
	Control + DAMGO	0,0076	**	F (3,7)=10.08, p<0.05
	Control + Fentanyl	0,0181	*	
	Control + Morphine	0,7648	ns	

Phosphorylation in untreated ΔGRK2/3 cells compared to agonist-induced phosphorylation in ΔGRK2/3 cells using one-way-ANOVA				
	**condition**	**p value**		**F**
**pT370-MOP**	ΔGRK2/3 untreated			
	ΔGRK2/3 + DAMGO	0,1936	ns	F (3,7)=4.29, p>0.05
	ΔGRK2/3 + Fentanyl	0,7011	ns	
	ΔGRK2/3 + Morphine	>0,9999	ns	
**pS375-MOP**	ΔGRK2/3 untreated			
	ΔGRK2/3 + DAMGO	0,0233	*	F (3,7)=6.01, p<0.05
	ΔGRK2/3 + Fentanyl	0,0134	*	
	ΔGRK2/3 + Morphine	0,1301	ns	
**pT376-MOP**	ΔGRK2/3 untreated			
	ΔGRK2/3 + DAMGO	0,2037	ns	F (3,7)=1.61, p>0.05
	ΔGRK2/3 + Fentanyl	0,8300	ns	
	ΔGRK2/3 + Morphine	0,9763	ns	
**pT379-MOP**	ΔGRK2/3 untreated			
	ΔGRK2/3 + DAMGO	0,4226	ns	F (3,7)=0.87, p>0.05
	ΔGRK2/3 + Fentanyl	0,7807	ns	
	ΔGRK2/3 + Morphine	0,9982	ns	

Phosphorylation in untreated ΔGRK5/6 cells compared to agonist-induced phosphorylation in ΔGRK5/6 cells using one-way-ANOVA				
	**condition**	**p value**		**F**
**pT370-MOP**	ΔGRK5/6 untreated			
	ΔGRK5/6 + DAMGO	0,0119	*	F(3,7)=11.51, p<0.05
	ΔGRK5/6 + Fentanyl	0,0081	**	
	ΔGRK5/6 + Morphine	0,9114	ns	
**pS375-MOP**	ΔGRK5/6 untreated			
	ΔGRK5/6 + DAMGO	0,2750	ns	F (3,7)=1.38, p>0.05
	ΔGRK5/6 + Fentanyl	0,2804	ns	
	ΔGRK5/6 + Morphine	0,8200	ns	
**pT376-MOP**	ΔGRK5/6 untreated			
	ΔGRK5/6 + DAMGO	0,0003	***	F (3,7)=45.57, p<0.05
	ΔGRK5/6 + Fentanyl	0,0002	***	
	ΔGRK5/6 + Morphine	0,9930	ns	
**pT379-MOP**	ΔGRK5/6 untreated			
	ΔGRK5/6 + DAMGO	0,0122	*	F (3,7)=16.41, p<0.05
	ΔGRK5/6 + Fentanyl	0,0401	*	
	ΔGRK5/6 + Morphine	>0,9999	ns	

Phosphorylation in untreated ΔGRKQ cells compared to agonist-induced phosphorylation in ΔGRKQ cells using one-way-ANOVA				
	**condition**	**p value**		**F**
**pT370-MOP**	ΔGRKQ untreated			
	ΔGRKQ + DAMGO	>0,9999	ns	F(3,7)=0.44, p>0.05
	ΔGRKQ + Fentanyl	>0,9999	ns	
	ΔGRKQ + Morphine	>0,9999	ns	
**pS375-MOP**	ΔGRKQ untreated			
	ΔGRKQ + DAMGO	>0,9999	ns	F(3,7)=0.17, p>0.05
	ΔGRKQ + Fentanyl	>0,9999	ns	
	ΔGRKQ + Morphine	>0,9999	ns	
**pT376-MOP**	ΔGRKQ untreated			
	ΔGRKQ + DAMGO	>0,9999	ns	F (3,7)=0.42, p>0.05
	ΔGRKQ + Fentanyl	>0,9999	ns	
	ΔGRKQ + Morphine	>0,9999	ns	
**pT379-MOP**	ΔGRKQ untreated			
	ΔGRKQ + DAMGO	>0,9999	ns	F (3,7)=0.26, p>0.05
	ΔGRKQ + Fentanyl	>0,9999	ns	
	ΔGRKQ + Morphine	0,7442	ns	

Supplementary Table 2 Potency (pEC_{50}) and maximal effect (E_{max}) for DAMGO, morphine and fentanyl mGsi recruitment in control and GRK knock-out cells. Concentration response curves from Supplementary Figure 1 were analysed using a three-parameter fit (Materials and Methods). Values represent mean [CI] for pEC50. Emax is expressed as BRET ratio (R), % of control cells (%C) and % of DAMGO in corresponding cell line (%). * $p<0.05$ compared to control cells, unpaired T-test.

		Control	ΔGRK2/3	ΔGRK5/6	QGRK
DAMGO	pEC_{50}	7.61 [7.84-7.37]	7.53 [7.91-7.14]	7.61 [7.85-7.36]	7.26 [7.57-6.98]
	E_{max} (R, %C)	0.08 (100)	0.06 (86)	0.09 (135)	0.06 (88)
	E_{max} (%)	100	100	100	100
Morphine	pEC_{50}	6.89 [7.08-6.70]	6.99 [7.29-6.66]	6.85 [7.04-6.62]	6.82 [7.27-6.34]
	E_{max} (R, %C)	0.06 (100)	0.05 (83)	0.07 (117)	0.05 (83)
	E_{max} (%)	88	83	85	83
Fentanyl	pEC_{50}	7.76 [7.95-7.53]	7.69 [7.94-7.42]	7.54 [7.81-7.26]	7.24 [7.75-6.78]
	E_{max} (R, %C)	0.08 (100)	0.06 (75)	0.09 (112)*	0.06 (75)
	E_{max} (%)	135	100	100	100
