# Supplementary material for: Key phosphorylation sites for robust β-arrestin2 binding at the MOR revisited
Source: Commun Biol. 2024 Aug 2;7:933. doi: 10.1038/s42003-024-06571-1 (PMC11297201; doi:10.1038/s42003-024-06571-1)
Supplement: Supplementary file 3 — Description of Additional Supplementary Materials [file 42003_2024_6571_MOESM3_ESM.pdf]

Description of Additional Supplementary Files

File name: Supplementary Data

Description: all numerical data from figures
